# Supplementary material for: Mating and male pheromone kill Caenorhabditis males through distinct mechanisms
Source: eLife. 2017 Mar 14;6:e23493. doi: 10.7554/eLife.23493 (PMC5378475; doi:10.7554/eLife.23493)
Supplement: Supplementary file 1. — DOI: http://dx.doi.org/10.7554/eLife.23493.021 [file elife-23493-supp1.docx]

**Supplementary Table 1. Lifespan summary**

| **Genotype/condition** | **Mean LS ± std. error** | **% change** | **p value** | **N** | **Related Figure** |
| --- | --- | --- | --- | --- | --- |
| **Experiment 1** |  |  |  |  |  |
|  |  |  |  |  |  |
| 1m *fog-2* | 12.0 ± 0.4 | -- | -- | 40 | Fig. 1A |
| 2m *fog-2* | 10.6 ± 0.4 | -12% | 0.0397 | 40 | Fig. 1A |
| 4m *fog-2* | 9.9 ± 0.4 | -18% | 0.0012 | 60 | Fig. 1A |
| 8m *fog-2* | 7.7 ± 0.2 | -36% | <0.0001 | 80 | Fig. 1A |
|  |  |  |  |  |  |
| **Experiment 2** |  |  |  |  |  |
| 1h N2 | 12.3 ± 0.4 | -- | -- | 40 | Fig. 1B |
| 30h N2 | 12.0 ± 0.3 | +3% | 0.6436 | 58 | Fig. 1B |
|  |  |  |  |  |  |
| 1h masculinized herm | 12.4 ± 0.5 | -- | -- | 40 | Fig. 2C |
| 30h masculinized herm | 10.4 ± 0.3 | -16% | 0.0015 | 60 | Fig. 2C |
|  |  |  |  |  |  |
| **Experiment 3** |  |  |  |  |  |
| 1m *fog-2* | 13.8 ± 0.7 | -- | -- | 35 | Fig. 1C |
| 8m *fog-2* | 9.8 ± 0.5 | -29% | <0.0001 | 48 | Fig. 1C |
| 8m *daf-22* | 14.7 ± 0.7 | +7% | 0.4039 | 48 | Fig. 1C |
|  |  |  |  |  |  |
| 1m *daf-22* | 17.2 ± 0.6 | -- | -- | 35 | Fig. 1-S1A |
| 8m *daf-22* | 14.7 ± 0.7 | -15% | 0.0660 | 48 | Fig. 1-S1A |
|  |  |  |  |  |  |
| 1m+FUdR *daf-22* | 15.3 ± 0.3 | -- | -- | 35 | Fig. 1-S1A |
| 8m+FUdR *daf-22* | 14.7 ± 0.3 | -4% | 0.2117 | 48 | Fig. 1-S1A |
|  |  |  |  |  |  |
| **Experiment 4** |  |  |  |  |  |
| 1h *daf-22* | 14.2 ± 0.6 | -- | -- | 35 | Fig. 1D |
| 1h *daf-22* MCP(8m) | 14.8 ± 0.8 | +4% | 0.4356 | 35 | Fig. 1D |
|  |  |  |  |  |  |
| 1m *daf-22* | 19.7 ± 0.5 | -- | -- | 34 | Fig. 1E |
| 1m *daf-22* MCP(8m) | 13.1 ± 0.4 | -34% | <0.0001 | 33 | Fig. 1E |
|  |  |  |  |  |  |
| **Experiment 5** |  |  |  |  |  |
| 1m *fog-2* | 12.1 ± 0.6 | -- | -- | 30 |  |
| 1m *fog-2* MCP(8m) | 9.8 ± 0.4 | -19% | 0.0046 | 28 |  |
|  |  |  |  |  |  |
| 1h *fog-2* | 13.8 ± 0.7 | -- | -- | 30 |  |
| 1h *fog-2* MCP(8m) | 12.6 ± 0.9 | -9% | 0.5965 | 29 |  |
|  |  |  |  |  |  |
| **Experiment 6** |  |  |  |  |  |
| 1m *daf-22* ctrl | 23.0 ± 0.9 | -- | -- | 30 | Fig. 1F |
| 1m *daf-22* MCP(1m) | 17.3 ± 0.7 | -25% | <0.0001 | 29 | Fig. 1F |
| 1m *daf-22* MCP(8m) | 16.1 ± 0.6 | -30% | <0.0001 | 30 | Fig. 1F |
|  |  |  |  |  |  |
| **Experiment 7** |  |  |  |  |  |
| 30m *daf-22* ctrl | 12.7 ± 0.3 | -- | -- | 150 | Fig. 1G |
| 30m *daf-22* MCP(30m) | 11.0 ± 0.2 | -13% | <0.0001 | 150 | Fig. 1G |
|  |  |  |  |  |  |
| 30m *daf-22* FUdR | 14.9 ± 0.2 | -- | -- | 150 | Fig. 1G |
| 30m *daf-22* MCP(30m) +FUdR | 15.3 ± 0.2 | +3% | 0.2964 | 150 | Fig. 1G |
|  |  |  |  |  |  |
| **Experiment 8** |  |  |  |  |  |
| N2 30h ctrl | 14.7 ± 0.5 | -- | -- | 107 | Fig. 1-S1E |
| N2 30h MCP(60m) | 10.6 ± 0.3 | -28% | <0.0001 | 98 | Fig. 1-S1E |
|  |  |  |  |  |  |
| *glp-1* 30h ctrl | 16.9 ± 0.5 | -- | -- | 96 | Fig. 1-S1E |
| *glp-1* 30h MCP(60m) | 15.9 ± 0.6 | -6% | 0.8933 | 97 | Fig. 1-S1E |
|  |  |  |  |  |  |
| **Experiment 9 @26°C** |  |  |  |  |  |
| 1m *glp-1* | 12.7 ± 0.8 | -- | -- | 44 | Fig. 1H |
| 8m *glp-1* | 13.3 ± 0.8 | +5% | 0.699 | 56 | Fig. 1H |
|  |  |  |  |  |  |
| **Experiment 10** |  |  |  |  |  |
| 1m *daf-22* ctrl | 15.8 ± 0.9 | -- | -- | 25 | Fig. 1-S1F |
| 1m *daf-22* MCP(8wt m) | 11.8 ± 0.5 | -25% | 0.0002 | 25 | Fig. 1-S1F |
| 1m *daf-22* MCP(8*glp-1* m) | 13.4 ± 0.6 | -15% | 0.0339 | 25 | Fig. 1-S1F |
|  |  |  |  |  |  |
| **Experiment 11** |  |  |  |  |  |
| Masculinized 1h | 12.3 ± 0.3 | -- | -- | 96 | Fig. 2A |
| Masculinized 1h MCP(8m) | 9.6 ± 0.3 | -22% | <0.0001 | 56 | Fig. 2A |
|  |  |  |  |  |  |
| **Experiment 12** |  |  |  |  |  |
| Masculinized 30h | 9.8 ± 0.2 | -- | -- | 111 | Fig. 2D |
| Masculinized 30h + FUdR | 12.2 ± 0.3 | +24% | <0.0001 | 119 | Fig. 2D |
|  |  |  |  |  |  |
| **Experiment 13** |  |  |  |  |  |
| 1m *fog-2* | 13.1 ± 0.6 | -- | -- | 50 | Fig. 3A |
| 1m1h_6d *fog-2* | 8.3 ± 0.4 | -37% | <0.0001 | 34 | Fig. 3A |
| 1m1h_1.5d *fog-2* | 11.9 ±0.6 | -9% | 0.0988 | 38 |  |
| 2m *fog-2* | 11.4 ± 0.5 | -13% | 0.0149 | 48 |  |
| 4m *fog-2* | 9.3 ± 0.6 | -29% | <0.0001 | 48 |  |
|  |  |  |  |  |  |
| **Experiment 14** |  |  |  |  |  |
| 1m *fog-2* | 10.9 ± 0.6 | -- | -- | 35 | Fig. 3B |
| 1m1h_1d *fog-2* | 11.4 ± 0.6 | +5% | 0.3697 | 31 | Fig. 3B |
| 1m1h_2d *fog-2* | 9.0 ± 0.6 | -17% | 0.0325 | 30 | Fig. 3B |
| 1m1h_3d *fog-2* | 9.1 ± 0.6 | -17% | 0.0452 | 34 | Fig. 3B |
| 1m1h_4d *fog-2* | 7.9 ± 0.5 | -28% | 0.0002 | 32 | Fig. 3B |
| 1m1h_5d *fog-2* | 8.3 ± 0.4 | -24% | 0.0006 | 34 | Fig. 3B |
| 1m1h_6d *fog-2* | 6.8 ± 0.3 | -38% | <0.0001 | 33 | Fig. 3B |
|  |  |  |  |  |  |
| **Experiment 15** |  |  |  |  |  |
| 1m *fog-2* | 13.8 ± 0.7 | -- | -- | 35 | Fig. 3C |
| 1m1h_3d *fog-2* | 10.8 ± 0.6 | -22% | 0.0175 | 32 | Fig. 3C |
| 1m2h_3d *fog-2* | 11.6 ± 0.9 | -16% | 0.1435 | 33 | Fig. 3C |
| 1m3h_3d *fog-2* | 10.6 ± 0.8 | -23% | 0.0147 | 34 | Fig. 3C |
|  |  |  |  |  |  |
| **Experiment 16** |  |  |  |  |  |
| 1m *fog-2* | 10.5 ± 0.5 | -- | -- | 35 |  |
| 1m1h_3d *fog-2* | 6.6 ± 0.2 | -37% | <0.0001 | 33 |  |
| 1m2h_3d *fog-2* | 6.3 ± 0.2 | -40% | <0.0001 | 32 |  |
| 1m3h_3d *fog-2* | 6.4 ± 0.3 | -39% | <0.0001 | 31 |  |
|  |  |  |  |  |  |
| **Experiment 17** |  |  |  |  |  |
| 1m *fog-2* | 13.8 ± 0.7 | -- | -- | 35 | Fig. 3D |
| 1m3h_3d(D1-3) *fog-2* | 10.6 ± 0.8 | -23% | 0.0147 | 34 | Fig. 3D |
| 1m3h_3d(D6-8) *fog-2* | 10.8 ± 0.6 | -22% | 0.0022 | 37 | Fig. 3D |
|  |  |  |  |  |  |
| **Experiment 18** |  |  |  |  |  |
| 1m *fog-2* | 12.1 ± 0.6 | -- | -- | 32 | Fig. 4A |
| 1m1h_6d h=*daf-22* | 9.0 ± 0.4 | -26% | 0.0001 | 29 | Fig. 4A |
|  |  |  |  |  |  |
| **Experiment 19** |  |  |  |  |  |
| 1m *daf-22* | 13.8 ± 0.6 | -- | -- | 40 | Fig. 4B |
| 1m1h_6d *daf-22* | 7.4 ± 0.4 | -46% | <0.0001 | 34 | Fig. 4B |
|  |  |  |  |  |  |
| **Experiment 20** |  |  |  |  |  |
| 1m *fog-2* | 10.5 ± 0.5 | -- | -- | 35 | Fig. 4C |
| 1m3h_3d *fog-2* | 6.4 ± 0.3 | -39% | <0.0001 | 31 | Fig. 4C |
| 1m3h_3d+FUdR *fog-2* | 10.2 ± 0.4 | -3% | 0.7086 | 36 | Fig. 4C |
|  |  |  |  |  |  |
| **Experiment 21** |  |  |  |  |  |
| 1m *fog-2* | 13.8 ± 0.7 | -- | -- | 35 | Fig. 4-S1A |
| 1m1h_6d *fog-2* | 10.3 ± 0.6 | -25% | 0.0006 | 31 | Fig. 4-S1A |
| 1m+FUdR *fog-2* | 13.9 ± 0.4 | +1% | 0.4079 | 35 | Fig. 4-S1A |
| 1m1h_6d+FUdR *fog-2* | 13.6 ± 0.5 | -1% | 0.3992 | 34 | Fig. 4-S1A |
|  |  |  |  |  |  |
| **Experiment 22** |  |  |  |  |  |
| 1m *fog-2* | 13.8 ± 0.7 | -- | -- | 35 |  |
| 1m1h_6d *fog-2* | 10.3 ± 0.6 | -25% | 0.0006 | 31 |  |
| 1m+FUdR *fog-2* | 13.9 ± 0.4 | +1% | 0.4079 | 35 |  |
| 1m3h_6d+FUdR *fog-2* | 13.6 ± 0.5 | -1% | 0.3992 | 34 |  |
| 1m3h_3d *fog-2* | 10.6 ± 0.8 | -23% | 0.0147 | 34 |  |
| 1m3h_3d+FUdR *fog-2* | 14.3 ± 0.7 | +4% | 0.8740 | 32 |  |
|  |  |  |  |  |  |
| **Experiment 23 @26°C** |  |  |  |  |  |
| 1m *glp-1* | 8.0 ± 0.4 | -- | -- | 40 | Fig. 4D |
| 1m1h_6d *glp-1* | 7.2 ± 0.4 | -10% | 0.3178 | 40 | Fig. 4D |
|  |  |  |  |  |  |
| **Experiment 24 @25°C** |  |  |  |  |  |
| 1m *glp-1* | 11.1 ± 1.0 | -- | -- | 27 | Fig. 4-S1B |
| 1m1h_6d *glp-1* | 11.1 ± 0.5 | 0% | 0.9149 | 43 | Fig. 4-S1B |
|  |  |  |  |  |  |
| **Experiment 25 @26°C** |  |  |  |  |  |
| 1m *glp-1* | 9.6 ± 0.4 | -- | -- | 40 |  |
| 1m1h_6d *glp-1* | 8.8 ± 0.5 | -8% | 0.238 | 40 |  |
|  |  |  |  |  |  |
| **Experiment 26** |  |  |  |  |  |
| 1m *fog-2* (*L4440*) | 12.6 ± 0.7 | -- | -- | 25 | Fig. 4H |
| 1m1h_4d *fog-2* (*L4440*) | 8.8 ± 0.5 | -30% | 0.0001 | 33 | Fig. 4H |
|  |  |  |  |  |  |
| 1m *fog-2* *(unc-62i)* | 11.9 ± 0.8 | -- | -- | 25 | Fig. 4H |
| 1m1h_4d *fog-2 (unc-62i)* | 10.6 ± 0.5 | -11% | 0.1249 | 34 | Fig. 4H |
|  |  |  |  |  |  |
| **Experiment 27** |  |  |  |  |  |
| 1m *pqm-1* | 11.9 ± 0.5 | -- | -- | 25 | Fig. 4I |
| 1m1h_6d *pqm-1* | 11.0 ± 0.6 | -8% | 0.2782 | 29 | Fig. 4I |
|  |  |  |  |  |  |
| **Experiment 28** |  |  |  |  |  |
| 1m *pqm-1* | 15.6 ± 0.6 | -- | -- | 25 | Fig. 5F |
| 1m *pqm-1* MCP(8m) | 14.4 ± 0.6 | -7% | 0.1627 | 25 | Fig. 5F |
|  |  |  |  |  |  |
| **Experiment 29** |  |  |  |  |  |
| 1m *C. r.* | 31.4 ± 1.7 | -- | -- | 72 | Fig. 6A |
| 1m1f_6d *C. r.* | 15.7 ± 1.2 | -50% | <0.0001 | 28 | Fig. 6A |
|  |  |  |  |  |  |
| **Experiment 30** |  |  |  |  |  |
| 1m *C. e.* | 10.2 ± 0.6 | -- | -- | 35 | Fig. 6B |
| 1m1h_6d *C. e.* x *C. e.* | 7.4 ± 0.4 | -27% | 0.0001 | 35 | Fig. 6B |
| 1m1f_6d *C. r.* x *C. e.* | 7.4 ± 0.4 | -27% | 0.0003 | 35 | Fig. 6B |
|  |  |  |  |  |  |
| **Experiment 31** |  |  |  |  |  |
| 1m *C. r.* | 35.8 ± 2.0 | -- | -- | 34 | Fig. 6C |
| 1m *C. r*. MCP(8m*C.r.)* | 37.8 ± 1.2 | +6% | 0.8501 | 34 | Fig. 6C |
|  |  |  |  |  |  |
| 1f *C. r.* | 27.6 ± 2.2 | -- | -- | 24 | Fig. 6D |
| 1f *C. r*. MCP(8m*C.r.)* | 27.0 ± 2.5 | -2% | 0.8306 | 30 | Fig. 6D |
|  |  |  |  |  |  |
| **Experiment 32** |  |  |  |  |  |
| *C.r.* 30f ctrl | 15.8 ± 0.9 | -- | -- | 60 | Fig. 6E |
| *C.r.* 30f MCP(30m*C.r.*) | 19.5 ± 1.3 | +23% | 0.0636 | 30 | Fig. 6E |
| *C.r.* 30f MCP(30m*C.e.*) | 18.5 ± 0.9 | +17% | 0.1770 | 60 | Fig. 6E |
|  |  |  |  |  |  |
| **Experiment 33** |  |  |  |  |  |
| *C.e.* 30 herm ctrl | 14.4 ± 0.8 | -- | -- | 90 | Fig. 6G |
| *C.e.* 30 herm MCP(30m*C.e*.) | 10.9 ± 0.6 | -24% | 0.0004 | 60 | Fig. 6G |
| *C.e.* 30 herm MCP(30m*C.r*.) | 11.9 ± 0.5 | -17% | 0.0042 | 90 | Fig. 6G |
|  |  |  |  |  |  |
| **Experiment 34** |  |  |  |  |  |
| 1m *C. brenneri* | 18.1 ± 0.8 | -- | -- | 33 | Fig. 7A |
| 1m *C. brenneri* MCP(8m*C.bn*.) | 17.8 ± 1.1 | -2% | 0.9915 | 32 | Fig. 7A |
|  |  |  |  |  |  |
| **Experiment 35** |  |  |  |  |  |
| 1m *C. nigoni* | 15.3 ± 0.4 | -- | -- | 32 | Fig. 7B |
| 1m *C. nigoni* MCP(8m*C.nigoni*) | 15.2 ± 0.6 | -1% | 0.7443 | 40 | Fig. 7B |
|  |  |  |  |  |  |
| **Experiment 36** |  |  |  |  |  |
| 1m *C. briggsae* | 13.7 ± 0.8 | -- | -- | 38 | Fig. 7C |
| 1m *C. briggsae* MCP(8m*C.br.*) | 10.3 ± 0.3 | -25% | 0.0192 | 54 | Fig. 7C |
|  |  |  |  |  |  |
| **Experiment 37** |  |  |  |  |  |
| 1m *C. tropicalis* | 17.7 ± 0.8 | -- | -- | 40 | Fig. 7D |
| 1m *C. tropicalis* MCP(8m*C.tr.*) | 12.2 ± 1.0 | -31% | 0.0002 | 60 | Fig. 7D |
|  |  |  |  |  |  |
| **Experiment 38** |  |  |  |  |  |
| 1m *C. remanei* | 36.0 ± 1.0 | -- | -- | 40 | Fig. 6F |
| 1m *C. remanei* MCP(8m *C.e*.) | 36.0 ± 1.0 | -0% | 0.8217 | 38 | Fig. 6F |
|  |  |  |  |  |  |
| **Experiment 39** |  |  |  |  |  |
| 1m *fog-2* ctrl | 17.4 ± 0.7 | -- | -- | 34 | Fig. 8 – S2A |
| 1m *fog-2* aMCP(8m) | 13.9 ± 0.7 | -20% | <0.0001 | 39 | Fig. 8 – S2A |
| 1m *fog-2* eMCP(8m) | 11.1 ± 0.6 | -36% | <0.0001 | 40 | Fig. 8 – S2A |
|  |  |  |  |  |  |
| **Experiment 40** |  |  |  |  |  |
| 1m *fog-2* ctrl | 19.5 ± 0.9 | -- | -- | 41 | Fig. 8 – S2B |
| 1m *fog-2* aMCP(8m) | 13.4 ± 0.5 | -31% | <0.0001 | 40 | Fig. 8 – S2B |
| 1m *fog-2* eMCP(8m) | 11.8 ± 0.4 | -39% | <0.0001 | 40 | Fig. 8 – S2B |
|  |  |  |  |  |  |
| **Exp 39 + Exp 40** |  |  |  |  |  |
| 1m *fog-2* ctrl | 18.5 ± 0.6 | -- | -- | 75 | Fig. 8A |
| 1m *fog-2* aMCP(8m) | 13.9 ± 0.4 | -25% | <0.0001 | 79 | Fig. 8A |
| 1m *fog-2* eMCP(8m) | 11.4 ± 0.4 | -40% | <0.0001 | 80 | Fig. 8A |
|  |  |  |  |  |  |
|  |  |  |  |  |  |
