# Supplementary material for: Mating and male pheromone kill Caenorhabditis males through distinct mechanisms
Source: eLife. 2017 Mar 14;6:e23493. doi: 10.7554/eLife.23493 (PMC5378475; doi:10.7554/eLife.23493)
Supplement: Supplementary file 2. — DOI: http://dx.doi.org/10.7554/eLife.23493.022 [file elife-23493-supp2.docx]

**Supplementary Table 2. Body size measurements**

| **Genotype/**  **condition** | **N** |  | **Day 1** | **Day 2** | **Day 3** | **Day 4** | **Day 5** | **Day 6** | **Day 7** |
| --- | --- | --- | --- | --- | --- | --- | --- | --- | --- |
| ***fog-2*** unmated | 31 | **Body length ± SE (µm)** | 903.6 ± 5.0 | 961.9 ± 5.6 | 982.8 ± 7.2 | 976.2 ± 7.2 | 959.0 ± 7.4 | 935.9 ± 8.1 | 931.5 ± 10.3 |
| ***fog-2***  mated | 30 |  | 893.8 ± 5.1 | 938.2 ± 5.6 | 955.4 ± 7.1 | 941.0 ± 9.5 | 913.7 ± 14.0 | 855.9 ± 19.4 | 851.4 ± 24.5 |
| @20°C |  | **p value** | 0.1813 | 0.004 | 0.0088 | 0.0044 | 0.0055 | 0.0004 | 0.0015 |
|  |  | **% change** | -1.1% | -2.5% | -2.8% | -3.6% | -4.7% | -8.6% | -8.6% |
|  |  |  |  |  |  |  |  |  |  |
| ***glp-1(e2141)***  unmated | 40 | **Body length ± SE (µm)** | 789.6 ± 11.3 | 880.1 ± 6.4 | 894.0 ± 7.1 | 895.1 ± 7.3 | 888.8 ± 7.9 | 872.0 ± 8.8 | 874.3 ± 9.0 |
| ***glp-1 (e2141)***  mated | 40 |  | 789.6 ± 11.3 | 887.0 ± 5.9 | 892.7 ± 8.1 | 894.0 ± 6.7 | 888.1 ± 10.8 | 868.6 ± 12.0 | 857.5 ± 11.4 |
| @26°C |  | **p value** | - | 0.4282 | 0.9083 | 0.9093 | 0.9567 | 0.8173 | 0.2510 |
|  |  | **% change** | - | 0.8% | -0.1% | -0.1% | -0.1% | -0.4% | -1.9% |
|  |  |  |  |  |  |  |  |  |  |
| ***fog-2*** 1m control | 30 | **Body length ± SE (µm)** | 789.6 ± 11.3 | 914.5 ± 6.5 | 949.2 ± 9.8 | 970.7 ± 9.6 | 985.2 ± 10.6 | 960.0 ± 11.8 | 945.5 ± 11.1 |
| ***fog-2*** 1m MCP(8m) | 48 |  | 795.9 ± 10.9 | 917.5 ± 6.6 | 929.2 ± 12.3 | 954.3 ± 13.7 | 968.5 ± 6.1 | 965.5 ± 5.7 | 948.1 ± 10.6 |
| @20°C |  | **p value** | 0.2962 | 0.7546 | 0.2301 | 0.3708 | 0.1537 | 0.4829 | 0.8696 |
|  |  | **% change** | 0.8% | 0.3% | -2.1% | -1.7% | -1.7% | 0.6% | 0.3% |
