# Supplementary material for: Mating and male pheromone kill Caenorhabditis males through distinct mechanisms
Source: eLife. 2017 Mar 14;6:e23493. doi: 10.7554/eLife.23493 (PMC5378475; doi:10.7554/eLife.23493)
Supplement: Supplementary file 3. — DOI: http://dx.doi.org/10.7554/eLife.23493.023 [file elife-23493-supp3.docx]

**Supplementary Table 3. Mated males microarrays_SAM (0% FDR)**

**Up-regulated:**

| **Rank** | **Sequence Name** | **Gene Name** | **Ave. fold change** | **SAM score** | **Gene description** |
| --- | --- | --- | --- | --- | --- |
| 1 | F59D8.2 | vit-4 | 18.13 | 13.72 | vit-4 is predicted to have lipid transporter activity, based on protein domain information. |
| 2 | F59D8.1 | vit-3 | 35.02 | 13.09 | vit-3 encodes a vitellogenin, a precursor of the lipid-binding protein related to vertebrate vitellogenins and mammalian ApoB-100, a core LDL particle constituent (OMIM:107730); VIT-3 is a major yolk component, but as loss of VIT-3 activity via RNA-mediated interference (RNAi) does not result in any abnormalities, VIT-3 likely functions redundantly with other vitellogenins to provide essential nutrients to the developing embryo; VIT-3 is expressed exclusively in the adult hermaphrodite intestine, from which it is secreted into the pseudocoelomic space and finally taken up by oocytes; in males, vit-3 expression may be negatively regulated by MAB-3, a DM binding domain-containing transcription factor required for male sexual development. |
| 3 | C04F6.1 | vit-5 | 13.99 | 11.49 | vit-5 encodes a vitellogenin, a lipid-binding protein precursor related to vertebrate vitellogenins and mammalian ApoB-100, a core LDL particle constituent; by homology, VIT-5 is predicted to function as a lipid transport protein; loss of vit-5 activity via large-scale RNA-mediated interference (RNAi) screens indicates that VIT-5 is required for embryogenesis and normal rates of postembryonic growth; VIT-5 is a major yolk component and is expressed exclusively in the adult hermaphrodite intestine from which it is secreted into the pseudocoelomic space and taken up by oocytes. |
| 4 | K12H6.5 | K12H6.5 | 6.62 | 10.59 |  |
| 5 | K07H8.6 | vit-6 | 21.26 | 10.35 | vit-6 encodes a vitellogenin precursor protein that is cleaved in the body cavity into two smaller yolk proteins, YP115 and YP88; in C. elegans, vitellogenin genes exhibit stage-, sex-, and tissue-specific expression being expressed exclusively in the adult hermaphrodite intestine. |
| 6 | F56H6.2 | F56H6.2 | 4.52 | 8.25 |  |
| 7 | Y46H3A.5 | Y46H3A.5 | 4.08 | 8.06 |  |
| 8 | C16C8.10 | C16C8.10 | 3.94 | 7.29 |  |
| 9 | F56D2.8 | F56D2.8 | 5.03 | 6.73 |  |
| 10 | F40G9.15 | F40G9.15 | 3.48 | 6.55 |  |
| 11 | C45G7.2 | ilys-2 | 2.85 | 6.28 | ilys-2 is involved in defense response to Gram-positive bacterium; ilys-2 is predicted to have lysozyme activity, based on protein domain information. |
| 12 | T10D4.7 | T10D4.7 | 4.01 | 6.27 |  |
| 13 | C42D8.2 | vit-2 | 13.96 | 6.22 | vit-2 encodes the vitellogenin homolog YP170; vit-2 is expressed in the adult hermaphrodite intestine and VIT-2 is secreted into the pseudocoelomic space before being taken up by developing oocytes; vit-2 expression is regulated in a sex-, stage-, and tissue-specific manner by the ELT-2/GATA and MAB-3 transcription factors. |
| 14 | C39B5.10 | C39B5.10 | 4.81 | 6.18 |  |

**Down-regulated:**

| **Rank** | **Sequence Name** | **Gene**  **Name** | **Ave. fold change** | **SAM score** | **Gene description** |
| --- | --- | --- | --- | --- | --- |
| 1 | F49E11.6 | scl-11 | -23.70 | -15.20 | scl-11 encodes a predicted extracellular protein that is a member of the C. elegans family of SCP/TAPS domain-containing proteins. |
| 2 | R04B5.6 | R04B5.6 | -8.11 | -10.76 | R04B5.6 encodes one of two C. elegans sorbitol dehydrogense orthologs; by homology the product of R04B5.6 is predicted to catalyze the reversible oxidation of sorbitol to fructose in the presence of NAD+; in the embryo, an R04B5.6::gfp fusion is expressed in pharyngeal cells and head neurons. |
| 3 | ZK355.3 | ZK355.3 | -15.78 | -10.63 |  |
| 4 | T13B5.5 | lips-11 | -9.76 | -9.88 | lips-11 is predicted to have hydrolase activity, based on protein domain information. |
| 5 | H10D18.2 | scl-12 | -5.82 | -9.38 | scl-12 encodes a predicted extracellular protein that is a member of the C. elegans family of SCP/TAPS domain-containing proteins. |
| 6 | T13B5.6 | lips-12 | -5.04 | -9.36 | lips-12 is predicted to have hydrolase activity, based on protein domain information. |
| 7 | Y6E2A.4 | Y6E2A.4 | -5.22 | -9.26 |  |
| 8 | K02E7.6 | K02E7.6 | -7.29 | -9.18 |  |
| 9 | F56D6.8 | F56D6.8 | -10.08 | -8.80 |  |
| 10 | W10G11.15 | clec-129 | -10.03 | -8.00 |  |
| 11 | F56D6.9 | F56D6.9 | -22.94 | -7.93 |  |
| 12 | F38B6.4 | F38B6.4 | -4.20 | -7.49 | F38B6.4 is an ortholog of human GART (phosphoribosylglycinamide formyltransferase, phosphoribosylglycinamide synthetase, phosphoribosylaminoimidazole synthetase); F38B6.4 is predicted to have phosphoribosylamine-glycine ligase activity, phosphoribosylformylglycinamidine cyclo-ligase activity, phosphoribosylglycinamide formyltransferase activity, and ATP binding activity, based on protein domain information. |
| 13 | Y22F5A.5 | lys-2 | -8.30 | -7.46 | lys-2 is one of ten C. elegans lysozyme genes; as such, lys-2 can be predicted to have a role in lysozymal function including immune function. |
| 14 | T01C3.11 | T01C3.11 | -4.25 | -7.33 |  |
| 15 | F46B3.14 | F46B3.14 | -4.45 | -7.16 |  |
| 16 | F45D11.4 | F45D11.4 | -4.15 | -6.70 | F45D11.4, with F45D11.2 and F45D11.3, encodes a nematode-specific protein that entirely consists of one large (~300-residue) 'domain of unknown function' (DUF684) that is found in several other C. elegans proteins; a transcription unit of either F45D11.4, F45D11.2, or F45D11.3 (genes of essentially identical sequence) has a natural nonsense transcript that is up-regulated in vivo by smg[-] mutations, indicating that at least one of these three genes is a natural substrate for SMG-mediated nonsense suppresssion; since several other natural mRNA substrates of SMG suppression (e.g., rpl-3, rpl-8, rpl-10a, rpl-12) have protein products that are involved in translation, F45D11.4 protein may may function in translation as well. |
| 17 | F58E10.7 | F58E10.7 | -4.01 | -6.33 |  |
| 18 | F32B4.6 | F32B4.6 | -7.31 | -6.19 | F32B4.6 is an ortholog of human ABHD11 (abhydrolase domain containing 11). |
| 19 | Y116A8C.44 | Y116A8C.44 | -5.04 | -6.12 |  |
| 20 | C52D10.1 | C52D10.1 | -2.97 | -6.09 |  |
| 21 | F36G9.7 | F36G9.7 | -3.71 | -6.06 |  |
| 22 | F45D11.15 | F45D11.15 | -7.75 | -6.04 |  |
| 23 | EGAP7.1 | dpy-3 | -2.52 | -6.04 | dpy-3 encodes a cuticular collagen; along with dpy-2, dpy-7, dpy-8, and dpy-10, dpy-3 is required postembryonically for annular furrow formation and/or maintenance; specifically, DPY-3 activity is required for proper assembly of the DPY-7 collagen into the mature extracellular matrix; during each cuticle synthetic period, dpy-3 mRNA is expressed approximately four hours prior to the secretion of new cuticle. |
| 24 | C17B7.12 | C17B7.12 | -3.47 | -6.04 |  |
| 25 | C32B5.9 | fbxc-7 | -4.85 | -6.03 |  |
| 26 | T13B5.7 | lips-13 | -5.67 | -5.99 | lips-13 is predicted to have hydrolase activity, based on protein domain information. |
| 27 | F35E8.10 | F35E8.10 | -3.41 | -5.95 |  |
| 28 | B0286.3 | B0286.3 | -3.43 | -5.95 | B0286.3 is an ortholog of human PAICS (phosphoribosylaminoimidazole carboxylase, phosphoribosylaminoimidazole succinocarboxamide synthetase); B0286.3 is predicted to have ATP binding activity, based on protein domain information. |
| 29 | T01B10.1 | grd-4 | -4.72 | -5.91 | grd-4 encodes a hedgehog-like protein, with an N-terminal signal sequence and a C-terminal Ground domain; the Ground domain is predicted to form a cysteine-crosslinked protein involved in intercellular signalling, and it has subtle similarity to the N-terminal Hedge domain of HEDGEHOG proteins; GRD-4 is weakly required for normal molting; GRD-4 is also required for normal adult alae formation, growth to full size, and locomotion; all of these requirements may reflect common defects in cholesterol-dependent hedgehog-like signalling or in vesicle trafficking. |
| 30 | T22B7.7 | T22B7.7 | -7.06 | -5.90 | T22B7.7 is an ortholog of human ACOT9 (acyl-CoA thioesterase 9). |
| 31 | C07A9.9 | C07A9.9 | -3.02 | -5.89 |  |
| 32 | C48B4.1 | C48B4.1 | -3.08 | -5.82 | C48B4.1 is an ortholog of human ACOX2 (acyl-CoA oxidase 2, branched chain) and ACOX1 (acyl-CoA oxidase 1, palmitoyl); C48B4.1 is predicted to have acyl-CoA dehydrogenase activity, acyl-CoA oxidase activity, and flavin adenine dinucleotide binding activity, based on protein domain information. |
| 33 | F45D11.1 | F45D11.1 | -5.05 | -5.73 |  |
| 34 | C03G6.5 | C03G6.5 | -2.73 | -5.70 |  |
| 35 | C10H11.5 | ugt-27 | -4.36 | -5.65 | ugt-27 is an ortholog of human UGT3A2 (UDP glycosyltransferase 3 family, polypeptide A2) and UGT3A1 (UDP glycosyltransferase 3 family, polypeptide A1); ugt-27 is predicted to have transferase activity, transferring hexosyl groups, based on protein domain information. |
| 36 | C09C7.1 | zig-4 | -2.52 | -5.65 | zig-4 encodes a predicted secreted protein that is a member of the immunoglobulin superfamily of proteins; ZIG-4 activity is required for maintenance of ventral nerve cord organization: the AVKL/R and PVQL/R axons of the left and right ventral nerve cords do not maintain their proper spatial positions and drift into the opposite cord; a zig-4::gfp reporter fusion is expressed in the PVT, ASK, BAG, and M2 neurons, with expression also seen during the L1 stage in pharyngeal mesoderm and ectoderm. |
| 37 | F18E3.12 | F18E3.12 | -3.26 | -5.65 |  |
| 38 | C33G8.3 | C33G8.3 | -7.08 | -5.62 |  |
| 39 | C23G10.6 | C23G10.6 | -3.23 | -5.57 | C23G10.6 is an ortholog of human UGT3A2 (UDP glycosyltransferase 3 family, polypeptide A2) and UGT3A1 (UDP glycosyltransferase 3 family, polypeptide A1); C23G10.6 is predicted to have transferase activity, transferring hexosyl groups, based on protein domain information. |
| 40 | Y53F4B.32 | gst-29 | -2.79 | -5.54 | gst-29 is an ortholog of human HPGDS (hematopoietic prostaglandin D synthase). |
| 41 | Y32G9A.5 | Y32G9A.5 | -2.69 | -5.38 |  |
